# Cardiovascular risk factors and breast cancer incidence in a large middle-aged cohort study

**DOI:** 10.1186/s12885-022-09604-2

**Published:** 2022-05-12

**Authors:** Xiaoqi Zeng, Shanshan Jiang, Simin Ruan, Lijun Zhu, Huining Lian, Minfeng Liu, Zhaoze Guo, Jingyun Guo, Changsheng Ye, Yunjiu Cheng, Jianyu Dong

**Affiliations:** 1 Breast Center, Department of General Surgery, Nanfang Hospital, Southern Medical University, No.1838 North Guangzhou Avenue, Guangzhou, 510515 China; 2 Institute of Hematological Research, Shaanxi Provincial People’s Hospital, Xi’an, 710000 China; 3 Department of Ultrasonography, Nanfang Hospital, Southern Medical University, Guangzhou, 510515 China; 4 Department of Cardiology, The First Affiliated Hospital, Sun Yat-Sen University, Guangzhou, 510700 China

**Keywords:** Cardiovascular disease, Risk factors, Breast cancer, Cohort, Atherosclerosis risk in communities, Incidence, Menopause

## Abstract

**Background:**

Several studies have demonstrated that cardiovascular risk factors play a role in the etiology of breast cancer. However, the combined effect of cardiovascular risk factors on the risk of breast cancer is still uncertain.

**Methods:**

Data from the Atherosclerosis Risk in Communities (ARIC) study, a prospective cohort of middle-aged women, were used to investigate the association of individual and combined cardiovascular risk factors with breast cancer. Cox proportional hazards models were applied to calculate the hazard ratio (HR) and 95% confidence intervals (CI).

**Results:**

A total of 7501 women were included. During a mean follow-up of 19.7 years, 576 women were diagnosed with breast cancer. White women and premenopausal status were independently associated with increased risk of breast cancer. Of the individual cardiovascular risk factors, only obesity was independently associated with an increased risk of breast cancer (HR 1.29, 95% CI 1.04–1.61). Compared with women without cardiovascular risk factors, women having three or greater, but not those with fewer than three cardiovascular risk factors, had a significantly higher risk of developing breast cancer (HR 1.27, 95% CI 1.06–1.53). Subgroup analyses indicated that women with three or greater cardiovascular risk factors had higher risk of breast cancer among postmenopausal Black women, but not among premenopausal Black and White women.

**Conclusions:**

Combinations of cardiovascular risk factors are associated with increased risk of breast cancer in middle-aged women, especially in postmenopausal Black women. Joint interventions to modify cardiovascular risk factors could be used to prevent breast cancer in these higher-risk individuals.

## Background

Breast cancer is the most common female malignant tumor worldwide and comprises 30% of new diagnoses among women in the United States, with an estimated incidence of 268,600 in 2019 [1]. The “common soil” hypothesis suggests that breast cancer is commonly associated with pathogenetic mechanisms and predisposing conditions (intermediate phenotypes) or risk factors, which are strongly similar to other chronic degenerative disorders such as cardiovascular, cerebrovascular and neurodegenerative disease, as if they were emerging from the same piece of earth as distinct trees, but with intermingled roots [2]. Recent studies have indicated that breast cancer and cardiovascular disease share several common risk factors, which are potentially modifiable [3]. The American Heart Association (AHA) announced that ideal cardiovascular health should achieve accomplish the strategic impact goal of reducing deaths from cardiovascular diseases by 20% by 2020. It is believed that approximately 80% of cardiovascular diseases can be prevented through risk factor modifications, including current smoking habits, obesity, poor healthy diet score, poor physical activity, hypercholesterolemia, hypertension and diabetes [4]. There is accumulating evidence associating passive and active tobacco smoking and breast cancer risk [5]. Obesity is confirmed as an independent breast cancer risk factor, specifically in postmenopausal women [6]. Additionally, there is evidence that a high quality diet and lifestyle are associated with a lower likelihood of excess body weight, which can reduce risk of breast cancer [7]. Touvier et al. [8] demonstrates a modest but statistically significant inverse association between total cholesterol levels and breast cancer in the first meta-analysis of prospective studies. Moreover, a case-control study from Chile revealed that hypertensive women experience a 4 - fold increased risk of breast cancer [9] and some studies have reported that type 2 diabetes was associated with increased risk of breast cancer [10]. Much of the association between cardiovascular risk factors and breast cancer suggests a shared biology which may provide scope for better prevention, earlier detection, and safer treatment strategies including lifestyle modification and preventive treatment [11].

Considering the interaction in cardiovascular risk factors, it is possible that a combination of cardiovascular risk factors could convey more information than a single factor alone. The clinical effects of clustering of cardiovascular risk factors on breast cancer incidence still remain unknown. In addition, studies have found that women who experience a late menopause have an increased risk of developing breast cancer [12]. Together with the reported racial disparities in breast cancer [13], it remains unclear whether the association between cardiovascular risk factors and breast cancer risk could be modified by race/ethnicity and menopausal status.

To address this gap in knowledge, we used data from the Atherosclerosis Risk in Communities (ARIC) study and performed a secondary analysis to explore the relationship between independent and combining effects of cardiovascular risk factors and breast cancer in women from the United States (US) to evaluate the associations of risk factors across race/ethnicity and menopausal status.

## Methods

### Study population

**Fig. 1:**
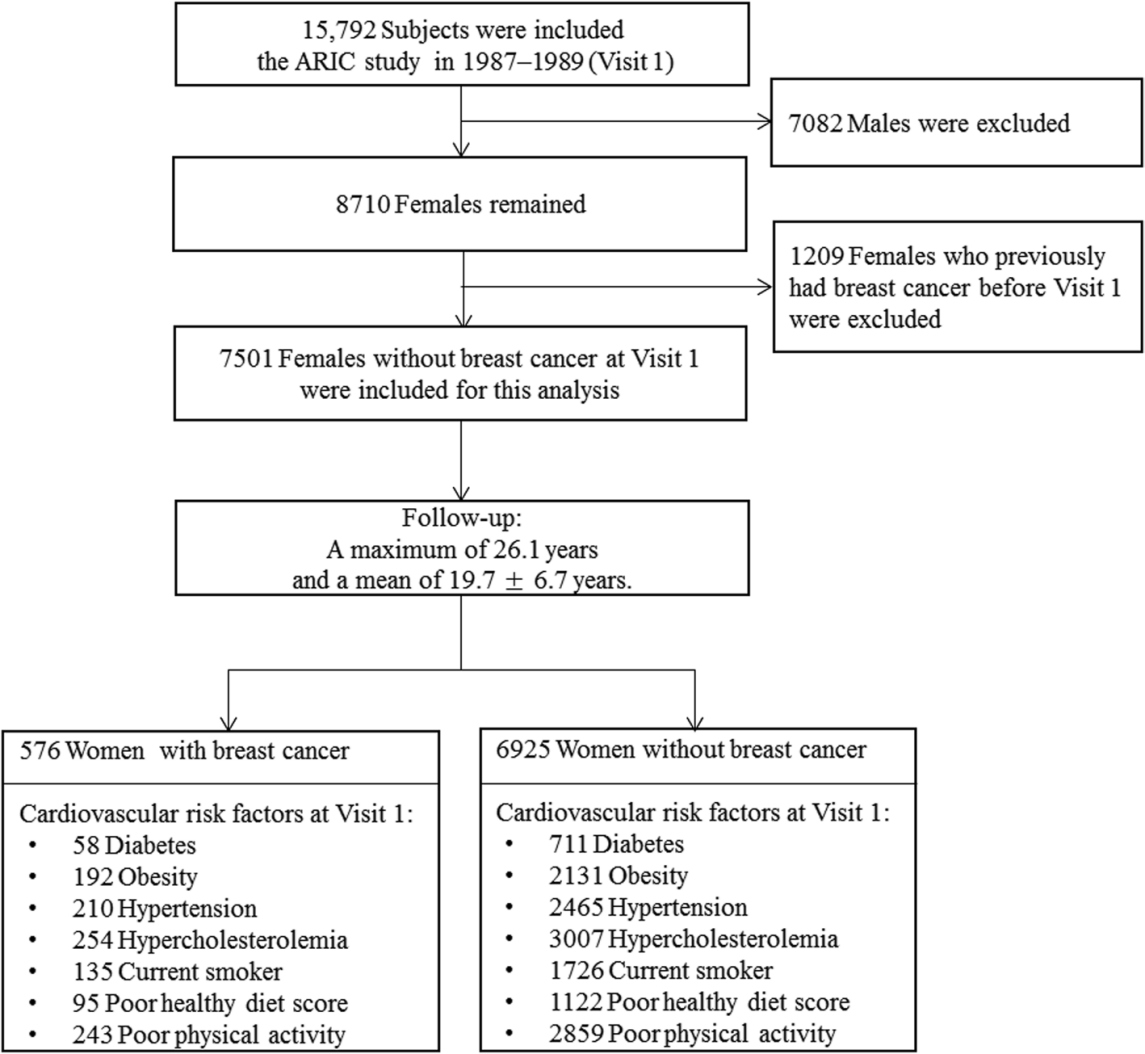
Study flow chart. The Atherosclerosis Risk in Communities (ARIC) Study is a prospective cohort study designed to investigate risk factors for atherosclerosis. A total of 15,792 individuals aged 45–64 years were recruited from 1987 to 1989 (Visit 1). We ultimately included 7501 women in this analysis and collected related data from Visit 1 to achieve a long-term follow-up

The ARIC Study is a prospective cohort study designed to investigate atherosclerosis risk factors among four communities in the United States (Forsyth County, NC; Jackson, MS; Suburbs of Minneapolis, MN; and Washington County, MD). The study objectives, design, sampling scheme, and cohort examination procedures have been described in detail [14]. In brief, between 1987 and 1989, each field center recruited and examined approximately 4000 subjects aged 45 to 64 years. African American residents were exclusively recruited in Jackson and oversampled in Forsyth County, whereas participants from the other two communities were predominantly white. Subsequently 15,792 individuals interviewed at home and then invited to a baseline clinical examination and were programmed for follow-up visits every three years (the second visit occurred in 1990–1992, the third in 1993–1995, the fourth in 1996–1998, and the fifth in 2011–2013). Participants were asked to fast for 12 hours before their morning clinic appointments. Fasting blood samples were drawn from an antecubital vein into vacuum tubes, and analyzed at ARIC centralized laboratories. Methods for blood collection and processing in ARIC have been described in detail [15]. Overall, 75% of potential participants responded to the home interview. Of these potential participants, 65% from the Jackson center and > 85% from the other three centers participated the baseline clinical examination. For the present analysis, we excluded 7082 males. We also excluded 1209 subjects who had been diagnosed with breast cancer before the recruitment questionnaire. After the above exclusions, 7501 subjects were included in this analysis (Fig. 1). The Institutional Review Board at each participating institution approved the study protocol and participants provided written informed consent before enrollment. We obtained the cohort data sets from the NIH Biologic Specimen and Data Repository Information Coordinating Center (BioLINCC) [16, 17].

### Baseline characteristics

Information regarding age, sex, race/ethnicity, education level, income, alcohol drinking habits, and menopausal status was obtained through interviews at Visit 1. Education level was classified into 3 categories: below high school, high school and graduate school, and education beyond graduate school. Income (US$ / Year) was classified into five groups: < 16,000, ≥16,000 to < 25,000, ≥25,000 to < 35,000, ≥35,000 to < 50,000, and ≥ 50,000. For the sub-analysis relative to alcohol drinking habits, participants were grouped into current, former, and never alcohol consumers. Menopausal status was defined as: primary amenorrhea, premenopause, perimenopause, natural postmenopause, and surgical post-menopause. We classified subjects who were premenopausal or perimenopausal at baseline into one category which we labeled “premenopause” and those with primary amenorrhea or natural and surgical menopause at baseline into a category labeled “postmenopause”.

### Cardiovascular risk factors

In this study, cardiovascular risk factors were defined using the American Heart Association definition, and included current smoker status, obesity, poor healthy diet score, poor physical activity, hypercholesterolemia, hypertension and diabetes [4]. (1) Smoking habit was self-reported as current, former, or never smoker at visit 1; (2) Body mass index (BMI) in kg/m^2^ was calculated based on measured weight wearing a scrub suit and standing height. The BMI was categorized into four groups: underweight (< 18.5), normal (≥18.5 to < 25.0), overweight (≥25.0 to < 30.0), and obese (≥30.0) [18]; (3) Poor healthy diet score was defined as having 0–1 components of a healthy diet score, assessed using the modified 66-item Harvard Food Frequency questionnaire [19]; (4) Poor physical activity was defined as no moderate or vigorous activity. Physical activity was assessed using the Baecke Physical Activity Questionnaire, which asked participants to report the frequency of involvement in up to four sports and walking in the previous year [20]. This was converted to minutes per week of moderate or vigorous physical activity; (5) Hypercholesterolemia was defined as total cholesterol >5.7 mmol/L. Fasting total cholesterol was measured by enzymatic methods [15]; (6) Hypertension was defined as systolic blood pressure ≥ 140 mmHg, diastolic blood pressure ≥ 90 mmHg, or use of antihypertensive medications. Blood pressure was taken three times in a seated position using a random-zero sphygmomanometer after a 5-min rest. The mean of the last two measurements was used for analysis [21]; (7) Diabetes was defined as a fasting glucose level of ≥7.0 mmol/L, a non-fasting level of ≥11.1 mmol/L, self-reported physician diagnosis of diabetes, or pharmacological treatment for diabetes. Impaired fasting glucose was defined as a fasting glucose level ranging 5.6–6.9 mmol/L. Fasting glucose was measured using the hexokinase/glucose-6-phosphate dehydrogenase method [15].

### Identification of incident breast cancer cases

Cases of incident breast cancer were ascertained from 1987 through 2013 by linkage to cancer registries and supplemented by hospital records. Primary site, date of cancer diagnosis, and source of diagnostic information were recorded. Information about breast cancer stage or histology was not collected.

### Statistical analysis

Data were analyzed using the statistical software Stata15.0 (STATA Corp, LLC., College Station, TX, USA). Statistical tests were two-sided and used a significance level of *P* < 0.05. Baseline characteristics and cardiovascular risk factors were compared between participants who did and did not develop breast cancer. Chi-square tests were performed for categorical variables and the Student’s *t*-test was used for continuous variables.

Cox regression was used to assess associations of cardiovascular risk factors and its components with breast cancer risk. Age was set as time scale. Follow-up time was defined as the time between the baseline examination until incident breast cancer, death, loss to follow-up, or last follow-up in 2013.

Individual cardiovascular risk factors were tested by treating each category as a categorical variable in the Cox models. We compared women with and without each individual cardiovascular risk factor at baseline. Models were constructed as follows: model 1 with no adjustments; model 2 adjusted for race/ethnicity (White, Black), menopausal status (premenopause, postmenopause); education level (below high school, high school and graduate school, and education beyond graduate school) and income (< 16,000, ≥16,000 to < 25,000, ≥25,000 to < 35,000, ≥35,000 to < 50,000, and ≥ 50,000). Model 3 was adjusted for variables in model 2 plus obesity status (underweight, normal, overweight and obesity), hypertension (yes, no), diabetes (yes, no), hypercholesterolemia (yes, no), smoking habit (current, former, never), healthy diet score (poor, non-poor) and physical activity (poor, non-poor).

In order to investigate the association between combinations of cardiovascular risk factors and breast cancer, we classified subjects as the obesity group (BMI ≥ 30.0) and the non-obesity group (BMI < 30.0). Moreover, we categorized subjects who were former and never smokers at baseline into one category which we labeled “non-current smoker” and those were current smokers at baseline into a category labeled “current smokers”. Restricted cubic splines with three knots were used to plot the association between breast cancer incidence and cardiovascular risk factors. Next, we calculated the hazard ratio (HR) and the 95% confidence interval (CI) of combinations of these cardiovascular risk factors (obesity, smoking, hypertension, diabetes, hypercholesterolemia, poor healthy diet score and poor physical activity) using models 1 and 2.

We also performed stratified analyses and then further calculated the association between individual and combined cardiovascular risk factors and breast cancer by race/ethnicity and menopausal status.

## Results

### Patient characteristics

After application of the exclusion criteria, a total of 7501 women were included in this study. The baseline characteristics stratified by breast cancer incidence during follow-up are shown in Table 1. Follow-up time was defined as the time between the baseline examination (Visit 1) until the incidence of breast cancer, death, loss to follow-up, or last follow-up in 2013. In this study cohort, 576 women were diagnosed with breast cancer (430 White and 146 Black). Compared to women without breast cancer, White women were more likely to develop breast cancer than Black women (*P* = 0.01). There were no differences between cases and non-cases with respect to baseline age, BMI, smoking habits, alcohol drinking habits, menopausal status, education level, income, healthy diet score and physical activity, or prevalence of hypertension, diabetes, and hypercholesterolemia. In addition, blood glucose levels and serum lipid levels were also not significantly different between women with and without breast cancer.

**Table 1:** Baseline characteristics of the participants by breast cancer incidence during follow-up

	Total	Breast Cancer	Non-Breast Cancer	
	***N*** = 7501	***N*** = 576	***N*** = 6925	***P***-Value
**Mean(SD)**				
**Age**	53.9 (5.7)	54.3 (5.8)	53.8 (5.7)	0.06
**BMI (kg/m**^**2**^**)**	27.9 (6.2)	28.0 (6.2)	27.9 (6.3)	0.61
**Waist-to-hip ratio**	0.9 (0.1)	0.9 (0.1)	0.9 (0.1)	0.75
**Blood glucose level (mmol/L)**	6.0 (2.5)	6.0 (2.3)	5.9 (2.5)	0.61
**TC (mmol/L)**	5.5 (1.4)	5.6 (1.2)	5.5 (1.4)	0.57
**HDL (mmol/L)**	1.5 (0.5)	1.5 (0.5)	1.5 (0.5)	0.95
**LDL (mmol/L)**	3.4 (1.2)	3.5 (1.1)	3.4 (1.2)	0.47
**TG (mmol/L)**	1.4 (0.9)	1.4 (0.7)	1.4 (1.0)	0.88
**ApoA1(g/L)**	1.4 (0.4)	1.4 (0.4)	1.4 (0.4)	0.47
**ApoB (g/L)**	0.9 (0.3)	0.9 (0.3)	0.9 (0.3)	0.32
**No. of subjects (%)**				
**Race/Ethnicity**				0.01*
White	5258	430 (74.7%)	4828 (69.7%)	
Black	2243	146 (25.3%)	2097 (30.3%)	
**Smoking habit**				0.60
Never	3973	305 (53.0%)	3668 (53.0%)	
Ever	1667	136 (23.6%)	1531 (22.1%)	
Current	1861	135 (23.4%)	1726 (24.9%)	
**Alcohol consumption**				0.62
Never	2677	207 (35.9%)	2470 (35.7%)	
Ever	1199	84 (14.6%)	1115 (16.1%)	
Current	3625	285 (49.5%)	3340 (48.2%)	
**Hypertension**				0.68
Yes	2675	210 (36.5%)	2465 (35.6%)	
No	4826	366 (63.5%)	4460 (64.4%)	
**Diabetes**				0.88
Yes	769	58 (10.1%)	711 (10.3%)	
No	6732	518 (89.9%)	6214 (89.7%)	
**Hypercholesterolemia**				0.75
Yes	3261	254 (44.1%)	3007 (43.4%)	
No	4240	322 (55.9%)	3918 (56.6%)	
**Healthy diet score**				0.86
Non-poor	6284	481 (83.5%)	5803 (83.8%)	
Poor	1217	95 (16.5%)	1122 (16.2%)	
**Physical activity**				0.67
Non-poor	4399	333 (57.8%)	4066 (58.7%)	
Poor	3102	243 (42.2%)	2859 (41.3%)	
**Menopausal status**				0.38
Premenopause	2121	172 (29.9%)	1949 (28.1%)	
Postmenopause	5380	404 (70.1%)	4976 (71.9%)	
**Education level**				0.61
Below High school	1793	137 (23.8%)	1656 (23.9%)	
High school/graduate school	3340	247 (42.9%)	3093 (44.7%)	
College/graduate/professional school	2368	192 (33.3%)	2176 (31.4%)	
**Income (US$/Year)**				0.53
< 16,000	2017	150 (26.0%)	1867 (27.0%)	
≥ 16,000 to < 25,000	1145	99 (17.2%)	1046 (15.1%)	
≥ 25,000 to < 35,000	1665	116 (20.1%)	1549 (22.4%)	
≥ 35,000 to < 50,000	1210	98 (17.1%)	1112 (16.0%)	
≥ 50,000	1464	113 (19.6%)	1351 (19.5%)	

* *P*-value < 0.05, statistically significant difference between women with and without breast cancer

Continuous variables presented as Mean (SD) and categorical variables presented as N (%)

After a maximum follow-up of 26.1 years and a mean of 19.7 ± 6.7 years, a total of 576 cases of breast cancer were reported (incidence rate = 3.9 per 1000 person-years). Table 2 shows the association among clinical factors for breast cancer incidence. Overall, only White women (HR 1.31, 95% CI 1.05–1.63) and premenopausal women (HR 1.50, 95% CI 1.24–1.81) were associated with an increased risk of breast cancer after adjusting for multiple factors. No significant associations were observed between serum lipids or blood glucose levels and breast cancer in either unadjusted or adjusted models. In addition, education level and income were also not significantly associated with the incidence of breast cancer.

**Table 2:** Hazard ratios (HRs) of clinical factors for breast cancer incidence

	Model 1 HR (95% CI)^**a**^	Model 2 HR (95% CI)^**b**^	Model 3 HR (95% CI)^**c**^
**Continuous variables**			
**BMI**	1.05 (0.96, 1.14)	1.08 (0.99, 1.18)	0.90 (0.75, 1.08)
**Waist-to-hip ratio**	0.99 (0.91, 1.07)	1.01 (0.93, 1.11)	0.94 (0.86, 1.04)
**Blood glucose level**	1.07 (0.98, 1.16)	1.09 (1.00, 1.19)*	1.09 (0.96, 1.23)
**TC**	0.97 (0.90, 1.06)	0.96 (0.89, 1.05)	0.99 (0.88, 1.12)
**HDL**	0.97 (0.90, 1.06)	0.97 (0.89, 1.05)	1.01 (0.92, 1.10)
**LDL**	0.99 (0.91, 1.07)	1.01 (0.92, 1.10)	1.01 (0.90, 1.13)
**TG**	1.01 (0.93, 1.10)	1.02 (0.94, 1.11)	0.98 (0.89, 1.08)
**ApolA1**	1.00 (0.92, 1.09)	1.00 (0.92, 1.09)	1.03 (0.95, 1.13)
**ApolB**	1.01 (0.93, 1.10)	1.04 (0.95, 1.13)	1.03 (0.93, 1.14)
**Categorical variables**			
**Waist-to-hip ratio**			
≤ 0.85	Reference	Reference	Reference
> 0.85	0.98 (0.82, 1.17)	1.02 (0.85, 1.23)	0.92 (0.75, 1.12)
**Blood glucose level (mmol/L)**			
< 5.6	Reference	Reference	Reference
≥ 5.6 to < 7.0	1.05 (0.87, 1.26)	1.08 (0.90, 1.30)	1.03 (0.85, 1.25)
≥ 7.0	1.17 (0.89, 1.55)	1.28 (0.96, 1.70)	1.18 (0.75, 1.85)
**TC (mmol/L)**			
Q1	Reference	Reference	Reference
Q2	0.92 (0.73, 1.16)	0.93 (0.73, 1.17)	0.93 (0.73, 1.17)
Q3	0.95 (0.76, 1.19)	0.99 (0.79, 1.26)	0.89 (0.61, 1.29)
Q4	0.83 (0.65, 1.05)	0.88 (0.69, 1.12)	0.75 (0.48, 1.18)
**HDL (mmol/L)**			
Q1	Reference	Reference	Reference
Q2	0.89 (0.70, 1.12)	0.88 (0.70, 1.11)	0.91 (0.72, 1.15)
Q3	0.94 (0.75, 1.18)	0.92 (0.73, 1.16)	1.00 (0.79, 1.26)
Q4	0.84 (0.67, 1.06)	0.83 (0.65, 1.04)	0.92 (0.72, 1.18)
**LDL (mmol/L)**			
Q1	Reference	Reference	Reference
Q2	0.97 (0.77, 1.22)	0.97 (0.77, 1.22)	0.95 (0.76, 1.21)
Q3	0.95 (0.75, 1.19)	0.98 (0.78, 1.23)	0.91 (0.70, 1.20)
Q4	0.86 (0.68, 1.09)	0.92 (0.72, 1.16)	0.82 (0.59, 1.15)
**TG (mmol/L)**			
Q1	Reference	Reference	Reference
Q2	1.04 (0.82, 1.33)	1.06 (0.83, 1.35)	1.04 (0.81, 1.32)
Q3	1.17 (0.93, 1.48)	1.23 (0.97, 1.56)	1.17 (0.91, 1.49)
Q4	1.11 (0.88, 1.42)	1.18 (0.92, 1.51)	1.07 (0.82, 1.41)
**Race/Ethnicity**			
Black	Reference	Reference	Reference
White	1.16 (0.96, 1.40)	1.18 (0.96, 1.46)	1.31 (1.05, 1.63)*
**Alcohol drinking habit**			
Never	Reference	Reference	Reference
Ever	0.96 (0.74, 1.23)	0.93 (0.72, 1.20)	0.91 (0.70, 1.18)
Current	1.06 (0.88, 1.27)	1.00 (0.82, 1.21)	0.99 (0.81, 1.22)
**Menopausal status**			
Postmenopause	Reference	Reference	Reference
Premenopause	1.47 (1.23, 1.77)*	1.49 (1.23, 1.79)*	1.50 (1.24, 1.81)*
**Education level**			
Below High school	Reference	Reference	Reference
High school/ graduate school	0.95 (0.77, 1.17)	0.90 (0.72, 1.12)	0.93 (0.74, 1.16)
College/ graduate/ professional school	1.08 (0.87, 1.35)	1.02 (0.80, 1.30)	1.09 (0.85, 1.40)
**Income (US$/Year)**			
< 16,000	Reference	Reference	Reference
≥ 16,000 to < 25,000	1.13 (0.88, 1.46)	1.08 (0.83, 1, 41)	1.10 (0.84, 1.44)
≥ 25,000 to < 35,000	0.92 (0.72, 1.17)	0.84 (0.65, 1.09)	0.87 (0.67, 1.13)
≥ 35,000 to < 50,000	1.09 (0.84, 1.40)	0.96 (0.72, 1.27)	1.00 (0.75, 1.33)
≥ 50,000	1.08 (0.85, 1.38)	0.89 (0.67, 1.19)	0.95 (0.71, 1.27)

Hazard ratios for continuous variables are expressed as unit change per 1 SD

^a^ Model 1 adjusted for no factors

^b^ Model 2 adjusted for race/ethnicity, menopausal status, education level, and income

^c^ Model 3 adjusted for variables in model 2 plus cardiovascular risk factor (Obesity status, hypertension, diabetes, hypercholesterolemia, smoking habit, healthy diet scores, and physical activity)

* *P*-value < 0.05

### Individual cardiovascular risk factors and breast cancer incidence

Table 3 summarizes the HRs for breast cancer diagnosis for women with different individual cardiovascular risk factors. Obese women were more likely to have breast cancer than normal weight women (adjusted HR 1.29, 95% CI 1.04–1.61), however, other cardiovascular risk factors: smoking habits, hypertension, diabetes, hypercholesterolemia, poor healthy diet score, and poor physical activity were not individually associated with the incidence of breast cancer after adjusting for age and jointly adjusted for age, race/ethnicity, menopausal status, education level or income, or after further multivariable adjustments.

**Table 3:** Hazard ratios (HRs) of individual cardiovascular risk factors for breast cancer incidence

	Model 1 HR (95% CI)^**a**^	Model 2 HR (95% CI)^**b**^	Model 3 HR (95% CI)^**c**^
**Obesity status**			
Normal weight	Reference	Reference	Reference
Underweight	0.91 (0.40, 2.05)	0.97 (0.43, 2.19)	0.92 (0.41, 2.08)
Overweight	1.08 (0.88, 1.32)	1.14 (0.93, 1.40)	1.14 (0.92, 1.40)
Obesity	1.21 (0.99, 1.48)	1.32 (1.06, 1.63)*	1.29 (1.04, 1.61)*
**Hypertension**			
No	Reference	Reference	Reference
Yes	1.01 (0.85, 1.20)	1.09 (0.91, 1.31)	1.04 (0.86, 1.25)
**Diabetes**			
No	Reference	Reference	Reference
Yes	1.11 (0.85, 1.46)	1.19 (0.90, 1.57)	1.13 (0.85, 1.51)
**Hypercholesterolemia**			
No	Reference	Reference	Reference
Yes	0.94 (0.80, 1.11)	0.99 (0.84, 1.17)	0.98 (0.83, 1.16)
**Smoking habit**			
Never	Reference	Reference	Reference
Ever	1.06 (0.87, 1.30)	1.04 (0.85, 1.28)	1.06 (0.86, 1.29)
Current	1.08 (0.88, 1.32)	1.09 (0.89, 1.33)	1.12 (0.91, 1.38)
**Healthy diet score**			
Non-poor	Reference	Reference	Reference
Poor	1.07 (0.86, 1.33)	1.08 (0.86, 1.34)	1.07 (0.86, 1.34)
**Physical activity**			
Non-poor	Reference	Reference	Reference
Poor	1.11 (0.94, 1.31)	1.17 (0.99, 1.39)	1.14 (0.95, 1.35)

^a^ Model 1 no adjustments

^b^ Model 2 adjusted for race/ethnicity, menopausal status, education level, and income

^c^ Model 3 adjusted for variables in model 2 plus cardiovascular risk factor (Obesity status, hypertension, diabetes, hypercholesterolemia, smoking habit, healthy diet score, and physical activity)

* *P*-value < 0.05

### [combining cardiovascular risk factors and breast cancer incidence

**Fig. 2:**
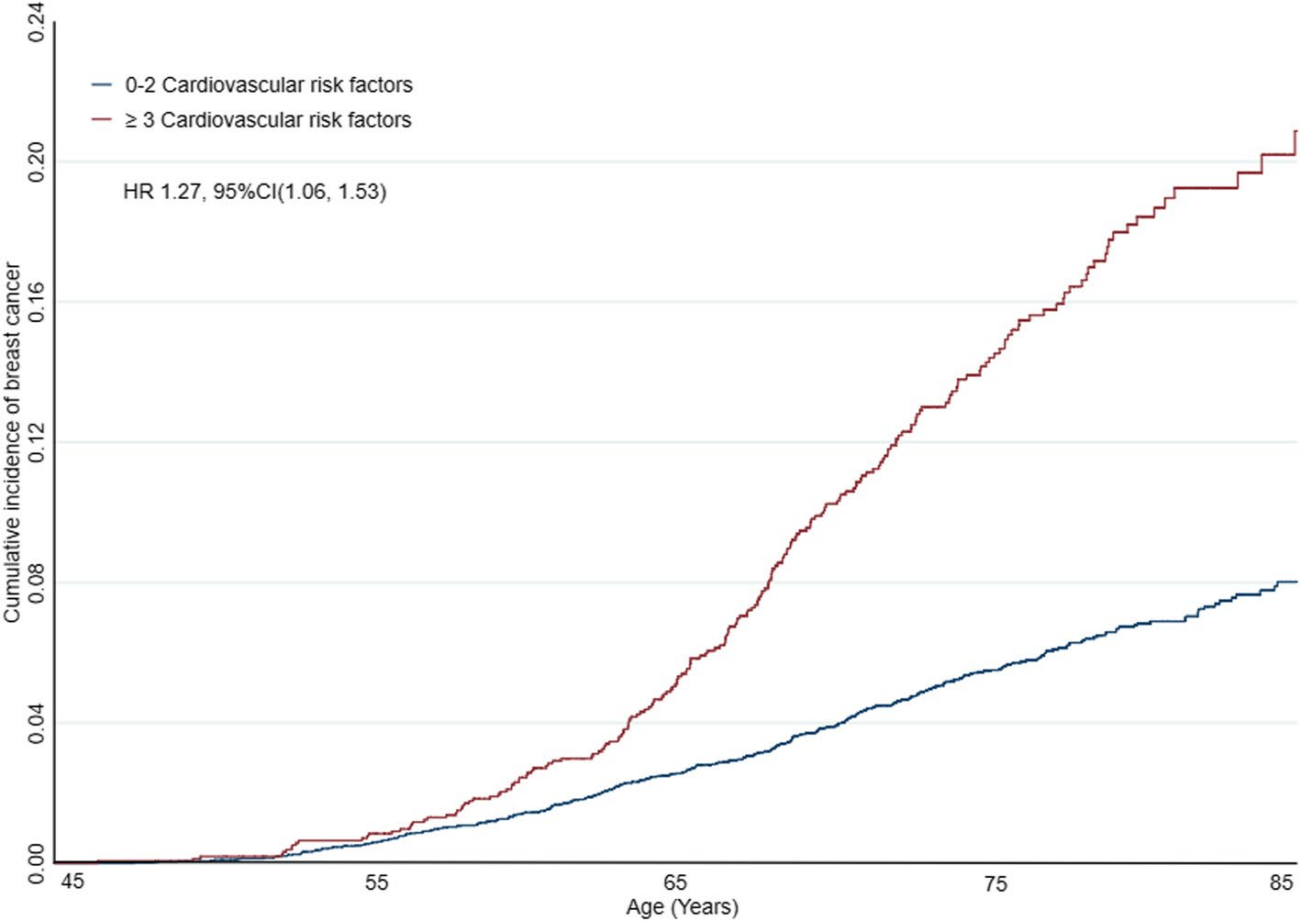
Kaplan-Meier curves for cumulative breast cancer incidence in subjects with a combination of cardiovascular risk factors. Shown is the association between a combination of cardiovascular risk factors and the incidence of breast cancer during the 23-year study period. Red lines indicate individuals having ≥3 cardiovascular risk factors. Compared with women without cardiovascular risk factors, women having ≥3 cardiovascular risk factors had a 27% higher cumulative breast cancer incidence (HR 1.27, 95% CI: 1.06–1.53, adjusted for race/ethnicity, menopausal status, education level, and income). However, no significant association was observed for women having < 3 cardiovascular risk factors

**Fig. 3:**
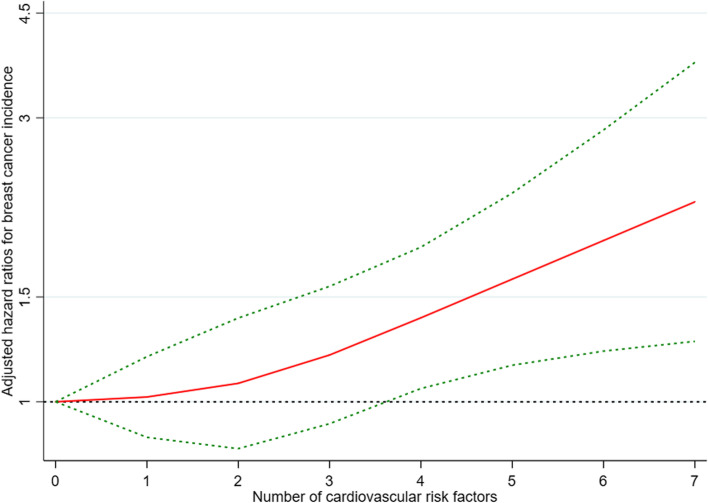
Restricted cubic spline for breast cancer incidence in subjects with a combination of cardiovascular risk factors. Shown is the dose-response relationship between a combination of cardiovascular risk factors and the incidence of breast cancer. Red lines indicate hazard ratios and green lines indicate 95% confidence intervals. Adjusted HRs are from the Cox proportional hazards model with adjustment for race/ethnicity, menopausal status, education level, and income

The graphical representation of the risk of breast cancer stratified by the number of cardiovascular risk factors is shown in Fig. 2. The cumulative risk curves for breast cancer continued to diverge during the follow-up period in subjects with three or greater cardiovascular risk factors. Figure 3 shows the dose-response association between the number of cardiovascular risk factors and breast cancer incidence, indicating that as the number of cardiovascular risk factors increased, the HR value increased. Table 4 shows the association of combined cardiovascular risk factors with breast cancer incidence. Compared to women with fewer than three risk factors, women with at least three cardiovascular risk factors had a higher risk of developing breast cancer. (unadjusted HR 1.15, 95% CI 0.97–1.36; adjusted HR 1.27, 95% CI 1.06–1.53).

**Table 4:** Hazard ratios (HRs) of combinations of cardiovascular risk factors in relation to breast cancer risk

Number of cardiovascular risk factors	Number of subjects	Model 1 HR (95% CI)^**a**^	Model 2 HR (95% CI)^**b**^
0–2	4877	Reference	Reference
≥ 3	2624	1.15 (0.97, 1.36)	1.27 (1.06, 1.53)*

^a^ Model 1 adjusted for no factor

^b^ Model 2 adjusted for race/ethnicity, menopausal status, education level, and income

* *P*-value < 0.05

### Stratified analyses of cardiovascular risk factors for breast cancer incidence

Considering that race/ethnicity and menopausal status might influence breast cancer incidence, we performed a stratified analysis of individual cardiovascular risk factors (Table 5) for breast cancer incidence. Among individual cardiovascular risk factors, a significant association remained among postmenopausal White women with obesity status and breast cancer risk (HR 1.49, 95% CI 1.11–2.01), while an inverse association was observed between postmenopausal Black women classified as overweight and breast cancer risk (HR 0.44, 95% CI 0.24–0.78). Moreover, current smokers experienced a higher risk of breast cancer than non-smokers among postmenopausal Black women (HR 1.88, 95% CI 1.19–2.96). In addition, women with poor healthy diet score experienced a higher risk of breast cancer (HR 1.61, 95% CI 1.02–2.54). However, no association was observed for other individual cardiovascular risk factors in the stratified analyses.

**Table 5:** Stratified analysis of hazard ratios (HRs) of individual cardiovascular risk factors for breast cancer incidence by race/ethnicity and menopausal status

	White		Black	
	Premenopause	Postmenopause	Premenopause	Postmenopause
	HR (95% CI)	HR (95% CI)	HR (95% CI)	HR (95% CI)
**Obesity status**				
Normal weight	Reference	Reference	Reference	Reference
Underweight	1.29 (0.18, 9.42)	0.70 (0.22, 2.21)	2.80 (0.27, 28.80)	1.00 (0.13, 7.48)
Overweight	1.47 (0.99, 2.18)	1.22 (0.92, 1.61)	2.24 (0.63, 7.92)	0.44 (0.24, 0.78)*
Obesity	0.95 (0.57, 1.57)	1.49 (1.11, 2.01)*	2.02 (0.57, 7.16)	0.86 (0.52, 1.42)
**Hypertension**				
No	Reference	Reference	Reference	Reference
Yes	0.96 (0.60, 1.52)	0.94 (0.73, 1.21)	1.82 (0.93, 3.54)	1.22 (0.81, 1.85)
**Diabetes**				
No	Reference	Reference	Reference	Reference
Yes	1.72 (0.81, 3.64)	0.86 (0.54, 1.39)	0.74 (0.27, 2.04)	1.46 (0.92, 2.31)
**Hypercholesterolemia**				
No	Reference	Reference	Reference	Reference
Yes	0.90 (0.61, 1.34)	0.96 (0.76, 1.20)	0.88 (0.43, 1.79)	1.22 (0.83, 1.79)
**Smoking habit**				
Never	Reference	Reference	Reference	Reference
Ever	1.11 (0.75, 1.65)	0.95 (0.72, 1.26)	0.74 (0.28, 1.97)	1.47 (0.90, 2.42)
Current	0.89 (0.55, 1.44)	1.03 (0.77, 1.37)	1.01 (0.48, 2.19)	1.88 (1.19, 2.96)*
**Healthy diet score**				
Non-poor	Reference	Reference	Reference	Reference
Poor	0.89 (0.54, 1.46)	0.96 (0.69, 1.33)	1.24 (0.57, 2.69)	1.61 (1.02, 2.54)*
**Physical activity**				
Non-poor	Reference	Reference	Reference	Reference
Poor	1.38 (0.96, 1.99)	1.13 (0.89, 1.44)	1.14 (0.58, 2.25)	0.95 (0.64, 1.41)

Adjusted for education level, income plus cardiovascular risk factor (obesity status, hypertension, diabetes, hypercholesterolemia, smoking habit, healthy diet score, and physical activity)

* *P*-value < 0.05

The associations between breast cancer and combined cardiovascular risk factors stratified by race/ethnicity and menopausal status are shown in Table 6. Among Black women, a significant association still remained among postmenopausal women with at least three cardiovascular risk factors (HR 2.02, 95% CI 1.32–3.11), but women having fewer than three cardiovascular risk factors did not present any significant association with breast cancer risk. In addition, a combination of cardiovascular risk factors was not associated with any risk of breast cancer risk in premenopausal Black women. Moreover, no significant association was observed for combined cardiovascular risk factors and breast cancer incidence among premenopausal White and postmenopausal women. Furthermore, we were surprised to find that postmenopausal women (2109/5380, 39.2%), in general, or Black women (678/2243, 50.0%) presented higher rates of three or more cardiovascular risk factors, than all premenopausal women (515/2121 24.3%) or White women (1367/5258, 26.0%).

**Table 6:** Stratified analysis of Hazard ratios (HRs) of combinations of cardiovascular risk factors for breast cancer incidence by race/ethnicity and menopausal status

Number of cardiovascular risk factors	White				Black			
	Premenopause		Postmenopause		Premenopause		Postmenopause	
	N	HR (95%CI)	N	HR (95%CI)	N	HR (95%CI)	N	HR (95%CI)
0–2	1357	Reference	2534	Reference	249	Reference	737	Reference
≥ 3	284	1.05 (0.66, 1.66)	1083	1.12 (0.87, 1.44)	231	1.48 (0.77, 2.84)	1026	2.02 (1.32, 3.11)*

Adjusted covariates included education level and income. *P-Value < 0.05

## Discussion

The present analysis of incident breast cancer risk in a large-scale community-based cohort demonstrated there was an increased risk of breast cancer among women presenting the following characteristics: White women, and premenopausal status in midlife. Considering individual cardiovascular risk factors, only obesity was independently associated with an increased risk of breast cancer incidence for all women. In addition, there was a significantly strong association between the number of cardiovascular risk factors and breast cancer incidence over a nearly 20-year follow-up, indicating that having at least three cardiovascular risk factors was associated with increased breast cancer risk for all women, although this risk increased among postmenopausal Black women. No such association was observed among White women. The finding that White women or premenopausal in midlife were more likely to develop breast cancer, is consistent with our current understanding [11]. Although menopausal status at diagnosis is more relevant to the incidence of breast cancer, one point that should be mentioned is that, in general, we consider late age at menopause associated with prolonged estrogen exposure, which may be related to the timing of onset of cardiovascular risk factors.

In the present study, among the cardiovascular risk factors evaluated, obesity was the only factor independently associated with an increased breast cancer risk, however, the association between individual cardiovascular risk factors and incident breast cancer is still contradictory. Herein, we demonstrated that there was an obvious strong association between obesity and increased breast cancer incidence. Recent studies have demonstrated that premenopausal women with a high BMI exhibit an inverse association with breast cancer risk, while postmenopausal women with a high BMI show a positive correlation with higher breast cancer risk. The latter study is consistent with our findings, which provide further evidence demonstrating that the impact of obesity on breast cancer risk varies based on menopausal status. A meta-analysis showed that an inverse association between physical activity and breast cancer risk can be subdivided into menopausal status, whereby a stronger association between physical activity and breast cancer risk was found for premenopausal women but such association was not been observed in the present study [22]. Similar results were obtained in another study showing no association between physical activity and breast cancer incidence in the ARIC study regardless of menopausal status [23]. Possible explanation for this null association was the subset of the premenopausal women was too small for the study. A recent study suggested breast cancer risk was lower in women with high compared to low prudent/healthy dietary patterns (OR 0.89, 95%CI 0.81–0.99), indicating women with high fruit and vegetable intake and those that included poultry, fish, low-fat dairy and whole grains in their diet were less likely to develop breast cancer [7]. Furthermore, the present study suggested Black postmenopausal women with poor healthy diet scores had a 61% higher risk of developing breast cancer, which consistent with a meta-analysis of large prospective cohort studies [24]. In addition, although smoking habit was not associated with a higher incidence of breast cancer in our study, the stratified analysis identified a positive association between current smoking status and breast cancer risk among postmenopausal Black women but no such association in postmenopausal White women, suggesting that the association between smoking and postmenopausal breast cancer was inconsistent across racial/ethnic groups [25]. However, a recent multiethnic cohort study reported that higher breast cancer risk related to smoking habits was similar across racial/ethnic groups (African Americans, Japanese Americans, Latinos, Native Hawaiians and White women). Thus, the implication of smoking status as a risk factor requires further study and elucidation. Moreover, our analysis of serum lipid levels and the incidence of breast cancer found that none of the lipid biomarkers considered in this study were associated with the incidence of breast cancer, which is in agreement with the findings of a prospective study from the Women’s Health Study [26], although some studies have indicated there is an inverse association between high-density lipoprotein cholesterol (HDL-C) levels and breast cancer risk and a positive association between low-density lipoprotein cholesterol (LDL-C) levels and breast cancer risk [27]. A stratified analysis of the lipid biomarkers was not performed in the present study while another study showed no association in the total population but inverse association between HDL-C and breast cancer incidence in postmenopausal women in the ARIC study [28]. Further study between lipid biomarker for breast cancer incidence according to the menopausal status was needed. With regard to diabetes, our study showed there was no association between a history of diabetes and the incidence of breast cancer, which is inconsistent with several studies [29]. One possibility for the conflicting results involves the pharmacological treatment of diabetes, which may influence the development of breast cancer, and breast cancer risk also varied across different races/ethnicities. Metformin use in women with diabetes was associated with a lower incidence of invasive breast cancer [30, 31]. The present study also found that blood glucose levels weakly associated with breast cancer risk, but this significant difference disappeared after adjusting for cardiovascular risk factors. Above all, this study suggested that individual cardiovascular risk factors had a limited impact on the occurrence of breast cancer and breast cancer is a consequence of the combination of multiple risk factors.

We were surprised to observe there was a significant trend for higher breast cancer incidence in patients exhibiting a large number of cardiovascular risk factors. The combined effects of cardiovascular risk factors on breast cancer incidence stratified by race/ethnicity and menopausal status, to our knowledge, have not been previously considered in the literature.

Indeed, the finding that combined effects may influence the incidence of breast cancer was consistent with a meta-analysis published in 2019, which suggested that the metabolic syndrome (i.e., defined as the presence of at least three of the following five components: abdominal obesity, high triglycerides, low HDL-C, high blood glucose, and high blood pressure) was associated with a significantly increased risk of breast cancer, particularly among postmenopausal women, although the studies examined mainly concerned Caucasian and Asian women [32]. Further, a large cross-sectional study revealed that fewer than 1% of adults from the United States present ideal levels of all seven cardiovascular health components: cardiovascular health behavior (diet, physical activity, BMI, smoking) or cardiovascular health factors (blood pressure, total cholesterol, fasting blood glucose, smoking), indicating that a large proportion of American adults exhibit poor and intermediate risk cardiovascular disease factors, and thus represent a large target population [33]. In another study, participants meeting the goals of six to seven ideal health metrics (2.7% of the population) had a 51% lower risk of all incident cancer but the association with breast cancer was not statistically significant (*P* for trend = 0.11) [34]. However, in the present study, instead of cardiovascular health components, we assessed the potential association between cardiovascular risk factors and breast cancer incidence, and consequently found that the presence of at least three cardiovascular risk factors increased the rate of breast cancer incidence, especially among postmenopausal Black women.

The current study provides evidence of the association between race/ethnicity and menopausal status in American women. The findings of the present study contradict those of our previous study and warrants further discussion. We found that White women, and women with premenopausal status in midlife are more likely to develop breast cancer, however, in the stratified analysis we demonstrated having at least three cardiovascular risk factors was associated with increased breast cancer risk in postmenopausal Black women instead of in White women. Several studies have reported that the prevalence of comorbidities (i.e., obesity, diabetes, hypertension, cardiovascular disease, and respiratory disease) is higher, particularly among Black women [35, 36]. Our study found a similar variation, showing that 50.0% of Black women and 39.2% of postmenopausal women exhibited ≥3 cardiovascular risk factors, while the prevalence among White women was 26.0% and among premenopausal women the prevalence was 24.3%. Thus, our findings show there is variability in cardiovascular risk factors among women incidentally diagnosed with breast cancer in terms of race/ethnicity and menopausal status.

We may hypothesize that the potential mechanism for these differences involves the presence of circulating estrogen and adipokine levels, which may act as potential mediators linking cardiovascular risk factors and breast cancer. Indeed, ovarian synthesis of endogenous hormones is confirmed to play a role in breast cancer, especially in premenopausal breast cancer, although endogenous hormones decelerate the progression of cardiovascular risk in premenopausal women [37]. Nonetheless, after menopause, due to the loss of direct effects of estrogen on the cardiovascular system, women are more likely to experience increased risk of cardiovascular disease. In addition, estradiol is produced primarily in adipose tissue, therefore postmenopausal women exhibiting cardiovascular risk factors, including obesity and hypercholesterolemia, tend to have higher circulating levels of estradiol [38]. Moreover, hyperinsulinemia may also promote breast cancer by increasing circulating estrogen levels, a factor that has been associated with breast cancer risk in postmenopausal women [39] and is also associated with the activation of insulin/IGF signaling, and dyslipidemia, which are commonly considered mechanisms involved in breast cancer. Conversely, adiponectin, which is produced exclusively or predominantly by adipose tissue, is considered a protective factor as it exerts anti-vascular, anti-inflammatory, antidiabetic, and insulin-sensitizing effects [40]. A growing number of studies have demonstrated a significant inverse association between serum adiponectin levels and breast cancer [41]. Moreover, researchers have reported that lower levels of high molecular weight adiponectin in postmenopausal women were significantly associated with a higher BMI, type 2 diabetes, hypertension, glucose, and insulin levels, and lower HDL-C levels (*P* < 0.01 for all) [42]. Thus, lower levels of high molecular weight adiponectin are associated with increased numbers of cardiovascular risk factors, and may play role in mechanisms underlying the association between cardiovascular risk factors and postmenopausal breast cancer. It follows, therefore, that circulating estrogen and adipokines may play a role in the association between combined cardiovascular risk factors and breast cancer, especially in postmenopausal Black women.

Based on the findings of our study, we recommend that individuals presenting numerous cardiovascular risk factors should receive more attention, appropriate screening, and careful follow-up, particularly among the Black women, as a preventative strategy for the occurrence of breast cancer and other diseases. In addition, caution should also be exercised in other countries, as the current results derived from a population sample from the United States.

The strengths of our study included the analysis of a population-based prospective cohort consisting of a long follow-up duration. We were able to obtain information about cardiovascular risk factors in the study designed to investigate atherosclerosis risk factors in four communities in the United States. However, this study has several limitations. Although the sample size was adequately powered, it was relatively small for the present study. We did not further explore the association between cardiovascular risk factors and breast cancer subtypes due to absence of histologic information. Second, baseline characteristic and cardiovascular risk factors were obtained through interviews at Visit 1, without considering behavior changes occurring during the follow-up period. Third, our results might be susceptible to residual confounding due to missing information for some covariates. Although the interaction only existed between cardiovascular risk factors and race/ethnicity, we still stratified menopausal status to explore the association of combined cardiovascular risk factors with breast cancer because estrogen exposure plays important role in breast cancer and cardiovascular risk factors.

## Conclusion

The novel findings in this study demonstrate that combinations of cardiovascular risk factors are associated with an increased risk of breast cancer in participants of the ARIC Study cohort, while the effect of individual cardiovascular risk factors is limited to obesity alone. Having ≥3 cardiovascular risk factors increased the rate of breast cancer by 27%, and was particularly significant among postmenopausal Black women in the United States. In a public health perspective, joint interventions aimed at modifying cardiovascular risk factors could be used to prevent breast cancer in these higher-risk individuals.

## Data Availability

All data generated or analysed during this study are included in this published article and its supplementary information files.

## Abbreviations

ARIC: Atherosclerosis Risk in Communities
HR: Hazard ratio
CI: Confidence intervals
TC: Total cholesterol
TG: Triglyceride
HDL-C: High-density lipoprotein cholesterol
LDL-C: Low-density lipoprotein cholesterol
Apo AI: Apolipoprotein AI
Apo B: Apolipoprotein B
BMI: Body mass index
CRF: Cardiovascular risk factors
